# Mechanisms of the Oral–Gut Microbiota Axis in Adverse Pregnancy Outcomes

**DOI:** 10.3390/microorganisms14071453

**Published:** 2026-07-01

**Authors:** Yijia Wang, Yi Liu

**Affiliations:** 1 Department of Periodontics, School of Stomatology, Capital Medical University, Beijing 100070, China; yijiawang1994@163.com; 2 Laboratory of Tissue Regeneration and Immunology, Department of Periodontics, Beijing Key Laboratory of Tooth Regeneration and Function Reconstruction, School of Stomatology, Capital Medical University, Beijing 100070, China

**Keywords:** adverse pregnancy outcomes, oral–gut microbiota axis, maternal microbiome, microbial dysbiosis, placenta

## Abstract

Adverse pregnancy outcomes (APOs), including preterm birth, preeclampsia, low birth weight, recurrent miscarriage, gestational diabetes mellitus, and fetal growth restriction, remain major threats to maternal and offspring health. Increasing evidence links the maternal microbiome to pregnancy health, but most studies have examined individual microbial niches rather than their interactions. The oral cavity and gut are anatomically and immunologically connected and form a bidirectional oral–gut microbiota axis through microbial trafficking, immune signaling, and metabolite-mediated feedback. Emerging studies suggested that oral dysbiosis, periodontal inflammation, and gut microbial remodeling were associated with APOs, although direct causal evidence in human pregnancy remains limited. This review summarizes pregnancy-related remodeling of the oral–gut microbiota axis, evaluates clinical and experimental evidence linking oral and gut dysbiosis to APOs, and discusses potential mechanisms, including microbial translocation, immune and inflammatory activation, metabolic remodeling, epigenetic regulation, and outer membrane vesicle-mediated signaling. Candidate biomarkers, probiotic and dietary intervention strategies, and current translational limitations are also discussed. Overall, the oral–gut microbiota axis offers a useful framework for understanding microbiome-associated APOs, but standardized sampling, longitudinal cohorts, and mechanistic validation are required before clinical application.

## 1. Introduction

Adverse pregnancy outcomes (APOs), including preterm birth (PTB), preeclampsia (PE), low birth weight (LBW), recurrent miscarriage, gestational diabetes mellitus (GDM), and fetal growth restriction (FGR), remain major threats to maternal, fetal, and neonatal health [1,2,3,4]. They impair perinatal outcomes and are associated with long-term risks of cardiovascular, metabolic, and neurodevelopmental disease in offspring [2,3,5]. Their etiology is multifactorial and involves genetic susceptibility, placental dysfunction, maternal metabolism, environmental exposures, infection, and inflammation [6,7,8]. With advances in high-throughput sequencing, metagenomics, and metabolomics, the maternal microbiome has received increasing attention as a potential contributor to pregnancy health and disease. Dysbiosis of the gut, oral, vaginal, and placental microbiota has been associated with PTB, PE, GDM, and other APOs [9,10,11,12]. However, most studies have evaluated individual microbial niches, leaving interactions among maternal microbial habitats insufficiently explored.

Among maternal microbial niches, the oral cavity and gut are particularly relevant because they harbor dense microbial communities and are anatomically and immunologically connected [13]. Oral microorganisms and inflammatory products can reach the gastrointestinal tract through saliva swallowing, mucosal leakage, or hematogenous dissemination, whereas gut dysbiosis may influence oral and periodontal conditions through circulating microbial metabolites, endotoxin-related signals, and systemic immune changes [14,15,16]. This reciprocal communication is referred to as the oral–gut microbiota axis. In non-pregnant populations, this axis has been implicated in systemic inflammatory and metabolic diseases, including type 2 diabetes and non-alcoholic fatty liver disease [17,18]. During pregnancy, endocrine, immune, and metabolic adaptations may further modify both oral and gut ecosystems, creating a pregnancy-specific context in which oral–gut communication may be biologically relevant.

Emerging evidence supports the plausibility of this framework in pregnancy, alt-hough direct causal evidence remains limited. A recent multi-omics study of first-trimester pregnant women showed that maternal periodontitis was associated with altered fecal microbiota and metabolites, suggesting that periodontal dysbiosis might exert distal effects through the oral–gut microbiota axis [19]. In a controlled murine model, periodontitis during pregnancy altered gut microbial composition and increased intestinal inflammation, supporting a possible oral-to-gut route under experimental conditions [20]. Other studies have associated pregnancy-related oral microbiome shifts and periodontal pathogens with APOs, including PTB and LBW [21,22,23]. Longitudinal multi-site microbiome profiling has further shown that microbial communities across maternal body sites vary during pregnancy and may be related to pregnancy outcomes [24]. Gut dysbiosis has also been linked to PE through bacterial translocation and immune dysregulation in human samples and animal models [25]. Together, these findings suggest that oral dysbiosis, gut dysbiosis, microbial translocation, inflammatory signaling, and microbial metabolites may contribute to an interconnected framework for microbiome-associated APOs.

Nevertheless, this evidence should be interpreted cautiously. Many human studies are observational or cross-sectional and therefore demonstrate associations rather than causation. Although animal models provide mechanistic support, their findings cannot be directly extrapolated to human pregnancy without longitudinal and translational validation. This review therefore does not propose the oral–gut microbiota axis as a proven cause of APOs. Rather, it used this axis as an integrative framework to organize current evidence linking maternal oral and gut dysbiosis with APOs.

Unlike previous reviews, which have focused mainly on the maternal microbiome as a whole or on individual microbial niches, this review highlights the oral–gut microbiota axis as an integrated framework for understanding microbiome-associated APOs. We emphasize that pregnancy-related endocrine, immune, metabolic, and gastrointestinal adaptations may modify both oral and gut microbial ecosystems and reshape their bidirectional communication. We also integrate evidence from human observational studies, animal models, and in vitro experiments to distinguish association from causation and to clarify five potential mechanistic pathways: microbial trafficking, immune and inflammatory activation, metabolic remodeling, epigenetic regulation, and outer membrane vesicle-mediated signaling. By linking oral dysbiosis, gut microbial remodeling, placental dysfunction, and translational challenges within one framework, this review aims to provide a focused basis for future longitudinal studies, standardized microbiome monitoring, and precision prevention strategies.

As a narrative review, this article is based on a targeted literature search rather than a formal systematic review. PubMed was used as the primary database, and the reference lists of eligible articles were manually screened to identify additional relevant studies. The search focused mainly on articles published within the past 10 years, with priority given to studies from the past 5 years; earlier studies were retained when they provided essential clinical, methodological, or mechanistic evidence. English-language original studies, clinical and epidemiological studies, animal and in vitro experiments, systematic reviews, meta-analyses, and mechanistic reviews were included if they addressed oral or gut microbiota, pregnancy-related microbial changes, APOs, mechanisms, biomarkers, or interventions. Duplicate records, conference abstracts, editorials or commentaries without primary or mechanistic information, articles without accessible full texts, and studies unrelated to pregnancy, oral/gut microbiota, or APOs were excluded. Thus, this review uses the oral–gut microbiota axis as an integrative framework to synthesize current evidence.

## 2. Dynamic Remodeling of the Oral–Gut Microbiota Axis During Pregnancy

### 2.1. Conceptual Framework of the Oral–Gut Microbiota Axis

The oral–gut microbiota axis describes anatomical, microbial, metabolic, and immunological communication between the oral cavity and the gastrointestinal tract. The oral cavity is a major reservoir of commensals and pathobionts, whereas the gut provides an extensive mucosal interface involved in nutrient metabolism, immune education, and barrier function. Although these sites differ substantially in ecological conditions, they are connected by continuous saliva swallowing, microbial trafficking along the gastrointestinal tract, mucosal immune communication, and circulating microbial metabolites [13,26].

In the oral-to-gut direction, oral microorganisms and their products are continuously introduced into the gastrointestinal tract through swallowed saliva, mucosal leakage, or hematogenous dissemination. Under physiological conditions, gastric acid, bile acids, intestinal barrier integrity, and colonization resistance limit the survival and persistence of many oral microbes in the gut [26,27]. However, oral dysbiosis and periodontitis may increase the burden of swallowed pathobionts and inflammatory products, thereby increasing the likelihood that oral microorganisms influence gut microbial ecology. Experimental and clinical studies have shown that periodontitis is associated with gut microbial dysbiosis and that saliva-derived microorganisms may contribute to intestinal microbial alterations [14,15,28].

In the gut-to-oral direction, regulation is more likely to occur through host-mediated feedback than through direct retrograde bacterial movement. Gut dysbiosis may influence systemic immune tone, endotoxin-related signaling, short-chain fatty acid metabolism, and trimethylamine N-oxide (TMAO) production [16,27,29]. These systemic changes might alter periodontal tissue immunity, gingival inflammation, and oral microbial homeostasis in turn [13,16]. Thus, the oral–gut axis should be viewed as a reciprocal regulatory network rather than a one-way route of bacterial translocation. In pregnancy, this bidirectional framework should be interpreted cautiously, because direct evidence for a complete oral–gut causal loop in human pregnancy remains limited, and many studies still examine oral and gut microbiota separately.

### 2.2. Pregnancy as a Physiological Modifier of the Oral–Gut Microbiota Axis

Estrogen and progesterone levels rise substantially during pregnancy, particularly during the second and third trimesters [30,31]. In the oral cavity, pregnancy-related hormonal and immune changes can increase gingival vascular permeability, modify gingival crevicular fluid composition, and influence local inflammatory responses [22,32]. These changes may contribute to pregnancy-associated gingivitis and shifts in periodontal microbial communities. Previous reviews have emphasized that endocrine and immune adaptations during pregnancy may reshape the oral microbiome and may partly explain the reported associations among oral dysbiosis, periodontal inflammation, and APOs [21,22]. However, because most available evidence was observational, these changes should be interpreted as pregnancy-associated microbial remodeling rather than direct proof that specific oral microorganisms cause APOs. The gut microbiome is also dynamically remodeled during pregnancy. Pregnancy-related changes in intestinal motility, nutrient metabolism, bile acid profiles, immune adaptation, and insulin sensitivity can affect gut microbial diversity and function. Recent studies have suggested that the maternal gut microbiota changes across gestation and may interact with host metabolism, inflammatory status, and immune regulation [9,31].

**Figure 1:**
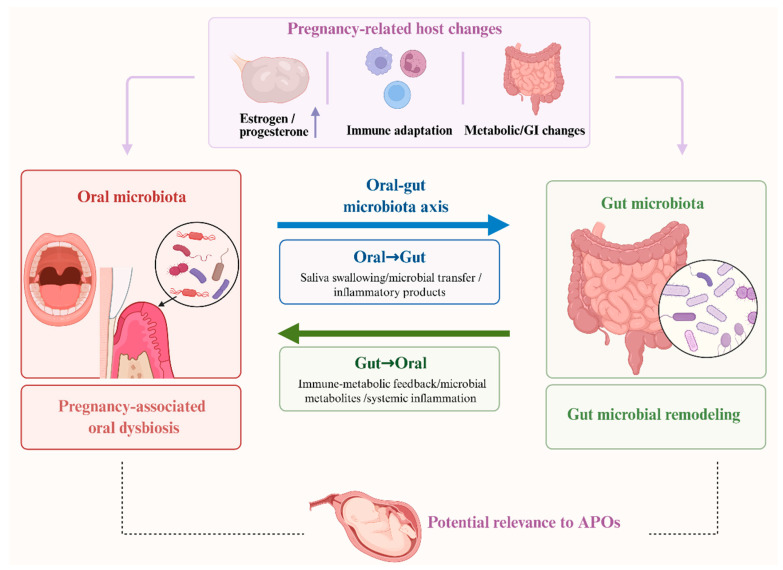
Pregnancy-related bidirectional regulation of the oral–gut microbiota axis and its potential link to APOs.

From the perspective of the oral–gut axis, pregnancy might simultaneously increase oral microbial and inflammatory exposure and altered the receptivity of the gut niche. For example, changes in gastrointestinal motility, mucosal immunity, and metabolic demand might affect whether swallowed oral microorganisms or inflammatory products are cleared, tolerated, or able to modify intestinal microbial ecology. Conversely, gut microbial changes might influence systemic inflammatory and metabolic signals that feed back to periodontal tissues. Figure 1 should therefore be interpreted as a conceptual model in which pregnancy-related endocrine and immune adaptations affect both oral and gut niches, while these niches might further regulate each other through microbial transfer, metabolites, and systemic immune signaling.

### 2.3. Microbial Alterations in Pregnancy and APOs: Evidence from Oral and Gut Microbiota

Building on the pregnancy-related oral and gut microbial remodeling described above, this section summarizes microbial alterations reported in oral and fecal samples across physiological pregnancy and APO-related clinical contexts. Because direct studies simultaneously profiling oral and gut microbiota in pregnant women remain limited, Table 1 and Table 2 are presented as complementary lines of evidence rather than proof of a complete oral–gut causal pathway. Table 1 summarizes pregnancy- and APO-related oral microbial alterations detected in subgingival plaque, supragingival plaque, and saliva. Most studies compared healthy pregnant women with non-pregnant women, whereas a smaller number examined pregnancy-associated periodontitis or APO-related outcomes. Table 2 summarizes fecal microbiota alterations reported in pregnancy-related disorders, including PE, GDM, pregnancy-induced hypertension (PIH), and FGR. Together, these tables indicate that both oral and gut microbial communities are altered in pregnancy-related contexts, but most available evidence remains observational and cannot establish causality.

More direct oral–gut bridging evidence is beginning to emerge. A multi-site first-trimester study linked maternal periodontitis with altered fecal microbiota and fecal metabolites in pregnant women [19], and an experimental pregnancy model showed that periodontitis altered gut microbial composition and increased intestinal inflammation [20]. These studies supported the biological plausibility of oral–gut axis-related pathways in APOs. However, a complete causal sequence from oral dysbiosis to gut remodeling and subsequent APO development in human pregnancy remains to be established.

## 3. Mechanistic Links Between the Oral–Gut Microbiota Axis and APOs

**Figure 2:**
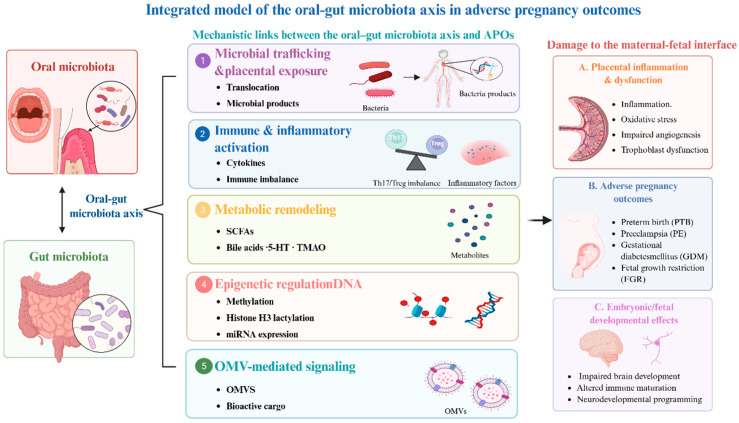
Mechanistic pathways linking the oral–gut microbiota axis to APOs.

Before discussing each pathway in detail, Figure 2 provides a conceptual overview of the proposed mechanisms linking the oral–gut microbiota axis to APOs. The figure summarizes five interconnected routes: microbial trafficking and placental exposure, immune and inflammatory activation, metabolic remodeling, epigenetic regulation, and outer membrane vesicle (OMV)-mediated signaling. These pathways should be interpreted as biologically plausible mechanisms supported by varying levels of evidence, rather than as fully established causal chains in human pregnancy.

### 3.1. Microbial Trafficking and Placental Exposure

Direct microbial trafficking should be considered at two levels. First, oral bacteria can enter the gastrointestinal tract through swallowed saliva, and large numbers of oral bacteria reach the gut under physiological conditions [26,27]. However, pregnancy-related changes in gastric acidity, gut motility, immune tolerance, and gut community structure might increase the likelihood that oral pathobionts or their products influence the intestinal niche [13,30,31].

Second, microbial presence at the maternal–fetal interface has been reported, but the level of evidence varies. Human studies have detected or isolated periodontal pathogens such as *P. gingivalis* and *Fusobacterium nucleatum* (*F. nucleatum*) in placental or pregnancy-related samples from cases of chorioamnionitis or preterm birth, supporting an association between these pathogens and APO-related tissues [43,44]. Animal models provided stronger mechanistic support: intravenous *F. nucleatum* can lead to placental colonization, inflammatory infiltration, and preterm birth-like outcomes in pregnant mice [45,46], and oral pathobionts such as *P. gingivalis* and *F. nucleatum* can survive gastrointestinal transit or colonize the gut in experimental models [47,48]. However, the full causal sequence from oral dysbiosis to gut disturbance, placental exposure, and APO development remains insufficiently demonstrated in human pregnancy.

### 3.2. Immune and Inflammatory Pathways

The oral–gut microbiota axis may also influence APOs indirectly through immune and inflammatory pathways. Periodontitis and gut dysbiosis can increase the systemic inflammatory burden even without direct bacterial translocation. Oral inflammatory lesions may release cytokines and inflammatory mediators, whereas gut dysbiosis can impair intestinal barrier function and promote lipopolysaccharide (LPS)-related inflammatory signaling [27,49]. These processes may contribute to systemic immune activation and alter the maternal–fetal immune environment.

One possible immune route involves T-cell imbalance. In experimental models, oral pathobionts can activate gut mucosal immune responses and promote pro-inflammatory Th17-related pathways, thereby disrupting immune homeostasis [15,50]. In human pregnancy, an altered Th17/Treg balance has been associated with PE and FGR, suggesting that systemic immune dysregulation may be relevant to APOs [51]. Nevertheless, these human findings remain largely associative and do not prove that oral or gut dysbiosis directly induces immune imbalance in APOs.

Maternal inflammation may further affect placental vascular and angiogenic pathways, although evidence should be considered according to study type. Human evidence is largely clinical and associative. In PE or FGR, angiogenic imbalance is often reflected by an increased sFlt-1/PlGF ratio or altered placental sFlt-1 and PlGF levels [52,53]. Large prospective studies also showed that circulating angiogenic factors can stratify the risk of severe PE and that placental sFLT1 contributes to the anti-angiogenic profile preceding PE [54,55]. These findings support a relationship among placental dysfunction, angiogenic imbalance, and APOs, but they do not alone prove that maternal inflammation directly impairs placental angiogenesis.

Animal evidence provides stronger experimental support for an inflammatory contribution. In mouse models, LPS-induced maternal inflammation increased maternal and placental pro-inflammatory cytokines and caused placental injury [56]. In inflammation- or hypoxia-induced murine PE models, placenta-targeted VEGF mRNA delivery improved PE-like features, supporting the relevance of angiogenic pathways in experimentally induced placental disease [57]. Studies further clarified cellular mechanisms: primary human trophoblasts exposed to hypoxia or hypoxia reoxygenation showed increased sFlt-1 secretion, reduced PlGF release, and a higher sFlt-1/PlGF ratio [58]. In addition, macrophage migration inhibitory factor, an inflammation-related mediator, could increase sFLT1 expression in trophoblast-based models and is associated with sFLT1 expression in preeclamptic placentas [59]. Together, these studies suggest that inflammatory signaling may contribute to placental angiogenic imbalance, but the causal relationship in human pregnancy remains incompletely established.

### 3.3. Metabolic Remodeling

Microbial metabolites provide another important link between oral–gut dysbiosis and APOs. The oral and gut microbiota can generate or regulate a wide range of metabolites, including SCFAs, amino acid derivatives, bile acid-related metabolites, amines, gases, and tryptophan–serotonin pathway intermediates. These metabolites may affect intestinal barrier function, maternal metabolism, immune regulation, and placental function.

Oral pathobionts and periodontal inflammation may influence gut metabolism directly or indirectly. For example, *P. gingivalis* can generate proteolytic metabolites, including SCFAs, amines, and gases, that may be ingested and modify gut microbiota composition by serving as alternative carbon and nitrogen sources. This process may promote the proliferation of harmful bacteria while inhibiting beneficial taxa [29,60]. In periodontitis-associated gut dysbiosis, altered SCFA metabolism may weaken intestinal barrier integrity and amplify inflammatory responses [61]. In pregnancy and early life, SCFAs are particularly relevant because they participate in maternal metabolism, immune regulation, and offspring immune programming [62,63,64]. Therefore, reduced SCFA production or altered SCFA signaling may represent a plausible metabolic pathway linking gut dysbiosis with APO-related inflammation and developmental outcomes.

Bile acid metabolism may also be involved in microbiota-associated APOs, as altered gut microbiota–bile acid interactions have been reported in intrahepatic cholestasis of pregnancy and GDM, suggesting a potential metabolic link between gut dysbiosis and pregnancy-related metabolic or inflammatory disturbances [65,66]. Other metabolic pathways also be involved. First-trimester disturbances in maternal tryptophan metabolism have been associated with embryonic and fetal growth parameters, suggesting that serotonin-related metabolic changes may be relevant to FGR [67]. However, most metabolomic studies remain associative, and it is often difficult to determine whether metabolite changes are causes of, consequences of, or compensatory responses to maternal metabolic and inflammatory alterations. Future longitudinal studies integrating oral, gut, serum, placental, and fetal metabolomic data are needed to define temporal and causal relationships.

### 3.4. Epigenetic Regulation

Inflammatory and metabolic signals derived from the maternal microbiota may also influence APOs through epigenetic regulation. Epigenetic mechanisms, including DNA methylation, histone modifications, and non-coding RNA regulation, can affect placental gene expression, fetal development, and long-term offspring health [68,69]. Because microbial metabolites and inflammatory mediators can influence epigenetic enzymes and chromatin states, the oral–gut microbiota axis may provide an upstream regulatory input into placental and fetal epigenetic programming.

Human studies suggested that APO-related inflammatory conditions were associated with altered epigenetic patterns in placental or fetal tissues. For example, early-onset PE has been associated with genome-scale hypomethylation in cord blood DNA [70]. Maternal periodontitis has also been discussed in relation to systemic disease susceptibility in offspring, including cardiovascular and allergic disease risks [71,72]. These studies supported an association between maternal inflammatory conditions, pregnancy complications, and epigenetic or long-term offspring outcomes, but they did not establish that oral–gut dysbiosis directly causes epigenetic remodeling in human pregnancy.

Mechanistic evidence linking microbiota to epigenetic programming was stronger in experimental models. Parental or maternal gut microbiota and microbiota-derived metabolites have been proposed to influence offspring’s epigenetic programming through the microbiome–metabolite–epigenome axis [73]. In a mouse study, parental microbiome differences were associated with altered offspring behavior, hippocampal DNA methylation, and gene expression, providing proof of principle that microbiota-related signals can shape fetal neurodevelopmental epigenetic patterns under experimental conditions [74]. Another mouse study showed that *P. gingivalis* administration during pregnancy increased histone H3 lactylation in fetal palatal tissues and interfered with palatal fusion through activation of the ADAM17 signaling pathway [75]. These findings indicated that oral pathobiont-related maternal exposure can affect fetal epigenetic or developmental pathways in experimental models. However, their relevance to human pregnancy required careful validation because dose, exposure route, host background, and placental structure differ between animal models and humans.

### 3.5. Outer Membrane Vesicles (OMVs)

Bacterial OMVs represent a distinct mechanism of host–microbe communication. These nanoscale vesicles are released by bacteria and can carry lipopolysaccharides, proteins, nucleic acids, enzymes, and other bioactive molecules. Because OMVs can deliver microbial signals without viable bacterial colonization, they may bridge microbial trafficking, immune activation, and metabolic disturbance. Current evidence suggests that OMV is a plausible mechanism by which oral or gut bacteria influence distant maternal or fetal tissues without whole-bacterium translocation. The representative studies summarized in Table 3 showed that bacterial OMVs can influence trophoblasts, fetal immune pathways, and brain development under experimental conditions, but direct evidence in human pregnancy remains insufficient. Future studies should determine whether oral or gut bacterial OMVs can be detected longitudinally in maternal blood, placenta, or fetal compartments during pregnancy; whether their cargo differs between uncomplicated and pathological pregnancies; and whether they causally contribute to placental inflammation, angiogenic imbalance, or fetal developmental abnormalities.

## 4. Translational Implications and Future Research Priorities

### 4.1. Candidate Signatures and Monitoring Strategies for the Oral–Gut Microbiota Axis During Pregnancy

As summarized in Table 1 and Table 2, pregnancy is accompanied by oral microbial remodeling, and several APOs are associated with gut microbial alterations. These findings provide a local microbial basis for oral–gut microbiota axis research. However, for clinical monitoring, local oral or fecal microbial changes alone may not fully reflect biological relevance to the maternal–fetal interface. Increasing attention has therefore been paid to candidate signatures detectable in amniotic fluid and maternal circulation, which may provide indirect evidence of oral/gut microbial trafficking or maternal microbiota–fetal communication. Table 4 summarizes representative amniotic fluid and serum signatures relevant to oral–gut microbiota axis-associated APO research. Table 4 highlights potential downstream or systemic signals. Together, these findings suggest that oral or gut dysbiosis may be reflected not only at local mucosal sites but also in pregnancy-related compartments more closely linked to the maternal–fetal interface.

Nevertheless, these signatures should be interpreted cautiously. At present, they are exploratory candidate markers rather than validated diagnostic biomarkers. For example, microbial EVs in amniotic fluid may support maternal microbiota–fetal communication, but they do not prove APO causation. Similarly, the detection of periodontal pathogens in amniotic fluid supports the possibility of microbial dissemination, but it does not determine whether the source is oral, hematogenous, gastrointestinal, or polymicrobial. Circulating SCFAs and TMAO-related metabolites may reflect gut microbial metabolism and maternal metabolic status, but they are also influenced by diet, liver and kidney function, host metabolism, gestational age, and pregnancy complications.

### 4.2. Intervention Strategies Targeting the Oral–Gut Microbiota Axis

Probiotics and dietary modulation have been explored as potential strategies for restoring oral and gut microbial balance during pregnancy, but current evidence supports cautious interpretation rather than universal recommendation. In the oral cavity, lozenges containing *Lactobacillus reuteri* (*L. reuteri*) have been reported to alleviate pregnancy gingivitis in a randomized controlled trial [84]. In a non-pregnant chronic periodontitis trial, *L. reuteri* used as an adjunct to scaling and root planing was associated with additional clinical improvement and greater reduction in *P. gingivalis* [85]. These findings supported the potential oral microbiota-modulating effect of *L. reuteri*. Regarding gut health, multi-strain probiotic formulas containing *Bifidobacterium* and *Lactobacillus* are generally considered safe during pregnancy, postpartum, and breastfeeding [86]. Some trials and meta-analyses suggested potential benefits for glycemic control, insulin resistance, inflammatory markers, and selected neonatal outcomes in women with GDM [87,88,89]. Maternal use of multi-species probiotics has also been associated with the partial restoration of offspring microbiota imbalances related to antibiotics or cesarean delivery [90].

However, probiotics’ efficacy remains inconsistent across studies. Differences in bacterial strains, strain combinations, dose, treatment duration, timing of administration, baseline diet, host metabolic status, antibiotic exposure, and outcome definitions make direct comparisons difficult. Current evidence therefore supported probiotics as promising but not yet standardized interventions; strain-specific, adequately powered randomized trials with harmonized endpoints and longer maternal–infant follow-up are still needed.

### 4.3. Current Limitations and Future Directions

Current research on the oral–gut microbiota axis and maternal–infant health faces several translational challenges. First, most studies remained cross-sectional, and few longitudinal cohorts collected oral, fecal, placental, amniotic fluid, and maternal clinical samples from preconception or early pregnancy through postpartum. Second, sampling methods were not standardized: studies differ in oral sampling sites, fecal collection procedures, gestational time points, storage conditions, DNA extraction methods, sequencing platforms, and bioinformatic pipelines. These differences may partly explain the limited reproducibility of microbial biomarkers across cohorts.

Third, mechanistic interpretation remains incomplete. Human studies have detected microbial or EVs signatures in pregnancy-related samples [80], while animal and in vitro models suggested that OMVs and inflammatory mediators can affect trophoblasts, immune responses, or fetal tissues [76,77,78,79]. These findings supported the biological plausibility of oral–gut microbiota axis-related mechanisms. However, they do not establish complete causal chains linking microbial translocation, OMV trafficking, metabolite remodeling, epigenetic regulation, and specific APOs in human pregnancy. Future mechanistic studies should therefore combine longitudinal human sampling with experimentally tractable models to clarify the timing, directionality, and causal relevance of these pathways.

Finally, clinical translation remains limited. Candidate microbial, EV, and metabolic signatures are still exploratory rather than validated diagnostic biomarkers, and probiotic findings remain inconsistent and appear to be strain-, dose-, timing-, and host-context-dependent. Future studies should prioritize multicenter longitudinal cohorts, standardized sampling and reporting frameworks, integrated microbiome–metabolome–immune profiling, spatial and single-cell technologies, and well-designed probiotic trials with harmonized clinical endpoints, strain-level characterization, safety assessment, and long-term maternal–infant follow-up.

## 5. Conclusions

In summary, the oral–gut microbiota axis provides an integrative perspective for understanding how maternal microbial ecosystems may be associated with APOs. During pregnancy, endocrine, immune, metabolic, and gastrointestinal adaptations can reshape both oral and gut microbial communities. Current evidence suggests that oral dysbiosis, periodontal inflammation, and gut microbial remodeling are repeatedly observed in pregnancy-related disorders, including PTB, PE, GDM, and FGR. However, most human studies remain observational, and these microbial changes should be interpreted as disease-associated signatures rather than established causal factors.

Several biological pathways may connect oral and gut dysbiosis with the maternal–fetal interface, including microbial trafficking and placental exposure, systemic immune and inflammatory activation, microbial metabolite remodeling, epigenetic regulation, and OMV-mediated signaling. Evidence from animal and in vitro models supports the plausibility of these mechanisms, particularly for bacterial translocation, trophoblast dysfunction, inflammatory imbalance, and vesicle-mediated host responses. Nevertheless, direct causal chains linking oral–gut microbial communication to specific APOs in human pregnancy remain insufficiently validated.

From a translational perspective, microbial and metabolic biomarkers from saliva, gingival plaque, feces, amniotic fluid, and maternal circulation may help improve early risk assessment. Probiotics, dietary modulation, and oral health management may also offer potential preventive strategies. At present, their clinical application is limited by inconsistent sampling methods, heterogeneous sequencing platforms, small or cross-sectional cohorts, and variable intervention outcomes. Future studies should prioritize standardized protocols, longitudinal multicenter cohorts, integrated microbiome–metabolome–immune profiling, and well-controlled intervention trials. These efforts will be essential for determining whether the oral–gut microbiota axis can be translated into reliable tools for APO prediction, prevention, and maternal–fetal health management.

## Figures and Tables

**Table 1:** Pregnancy- and APO-related alterations in oral microbiota.

Sample Type	Method	Reported Oral Microbiota Alteration(s)	Comparison	References
Human subgingival plaque	PCR	Higher prevalence of *Tannerella forsythia*; *P. gingivalis* positively correlated with progesterone levels	Healthy pregnant women vs. non-pregnant women	[33]
Human subgingival plaque	PCR	Increased: *P. gingivalis*, *Aggregatibacter actinomycetemcomitans*, and *Candida species*	Healthy pregnant women vs. non-pregnant women	[34]
Human supragingival plaque	16S rDNA sequencing	Increased: *Neisseria*, *Porphyromonas*, and *Treponema*	Healthy pregnant women vs. non-pregnant women	[35]
Human saliva	Spectrophotometry	Increased: *Streptococcus mutans*	Healthy pregnant women vs. non-pregnant women	[36]
Human saliva	16S rDNA sequencing	Increased: *P. nigrescens*	Healthy pregnant women vs. non-pregnant women	[37]
Human saliva	PCR	Increased: *Treponema denticola*, *Prevotella intermedia*, and *Fretibacterium* sp. *HOT360*; decreased: *Rothia dentocariosa*	Pregnant women with periodontitis vs. healthy pregnant women	[38]
Human saliva and subgingival plaque	PCR	Increased: *P. gingivalis* and *Tannerella forsythia* in subgingival plaque; *Prevotella intermedia* in saliva	TPL-PLBW vs. TPL-HD and healthy-HD groups	[39]

*P. gingivalis*, *Porphyromonas gingivalis*; TPL, threatened preterm labor; PLBW, preterm low birth weight; HD, healthy delivery.

**Table 2:** Gut microbiota alterations reported in pregnancy-related diseases.

Sample Type	Method	Reported Gut Microbiota Alteration(s)	Comparison	References
Human feces	16S rDNA sequencing	Increased: *Fusobacterium* and *Veillonella*; decreased: *Faecalibacterium* and *Akkermansia*	PE pregnant women vs. healthy pregnant women	[25]
Human feces	Metagenomic sequencing	Decreased: short-chain fatty acid (SCFA)-producing genera (*Faecalibacterium*, *Prevotella*, *Streptococcus*) and species (*Bacteroides coprophilus*, *Eubacterium siraeum*, *Faecalibacterium prausnitzii*, *Prevotella copri*, *Prevotella stercorea*)	GDM pregnant women vs. normal glucose tolerance	[40]
Human feces	Metagenomic sequencing	Increased: *Eubacterium rectale* and *Ruminococcus bromii*	PIH pregnant women vs. healthy pregnant women	[41]
Human feces	16S rRNA sequencing	Increased: *Lactobacillus (V32)* and *Catenibacterium (V38)*; decreased: *Ruminococcaceae (V22)*, *Bacteroides uniformis (V30)*, *Mollicutes RF39 (V52)*, and *Alistipes onderdonkii (V57)*	FGR pregnant women vs. healthy pregnant women	[42]

**Table 3:** Bacterial OMV-mediated responses relevant to pregnancy.

Bacterial OMV Source	Model	Main Reported Effect Relevant to APOs	References
*P. gingivalis*	Pregnant mice	Altered cortical neurons and tau phosphorylation in the fetal brain	[76]
*P. gingivalis*	Pregnant mice and trophoblast cells	Altered trophoblast metabolism and reduced fetal weight	[77]
*P.* *gingivalis*	Trophoblast cells	Disrupted trophoblast interactions involved in vascular transformation and immune homeostasis	[78]
*Helicobacter pylori*	Pregnant mice	Affected offspring thymic T-cell tolerance through maternal–fetal transmission	[79]

**Table 4:** Candidate amniotic fluid and serum signatures relevant to oral–gut microbiota axis-associated APO research.

Sample Type	Method	Candidate Signature(s)	Pregnancy or APO-Related Context	References
Amniotic fluid and fecal samples from pregnant women	16S rRNA sequencing, PCR, EV isolation, and proteomic analysis	Microbiota-derived EVs	Protein cargo of bacterial EVs in amniotic fluid and maternal feces showed similarities in origin and functional characteristics	[80]
Human amniotic fluid	PCR	*C. rectus*, *T. forsythia*, *P. gingivalis*, and *F. nucleatum*	Detection of periodontal pathogens in amniotic fluid was associated with PTB and/or LBW	[81]
Serum from pregnant women	Targeted metabolomics	SCFAs (gut microbiota-derived metabolites)	Altered circulating SCFA profiles were associated with pregnancy-related metabolic and inflammatory disorders, including GDM, PE, and intrahepatic cholestasis of pregnancy	[82]
Serum from pregnant women	Targeted metabolomics	TMAO	Altered maternal serum TMAO levels were associated with GDM	[83]

## Data Availability

No new data were created or analyzed in this study. Data sharing is not applicable to this article.
